# Crystal Structures of Histone and p53 Methyltransferase SmyD2 Reveal a Conformational Flexibility of the Autoinhibitory C-Terminal Domain

**DOI:** 10.1371/journal.pone.0021640

**Published:** 2011-06-28

**Authors:** Yuanyuan Jiang, Nualpun Sirinupong, Joseph Brunzelle, Zhe Yang

**Affiliations:** 1 Department of Biochemistry and Molecular Biology, Wayne State University School of Medicine, Detroit, Michigan, United States of America; 2 Advance Photon Source, Argonne National Lab, Argonne, Illinois, United States of America; McGill University, Canada

## Abstract

SmyD2 belongs to a new class of chromatin regulators that control gene expression in heart development and tumorigenesis. Besides methylation of histone H3 K4, SmyD2 can methylate non-histone targets including p53 and the retinoblastoma tumor suppressor. The methyltransferase activity of SmyD proteins has been proposed to be regulated by autoinhibition via the intra- and interdomain bending of the conserved C-terminal domain (CTD). However, there has been no direct evidence of a conformational change in the CTD. Here, we report two crystal structures of SmyD2 bound either to the cofactor product S-adenosylhomocysteine or to the inhibitor sinefungin. SmyD2 has a two-lobed structure with the active site located at the bottom of a deep crevice formed between the CTD and the catalytic domain. By extensive engagement with the methyltransferase domain, the CTD stabilizes the autoinhibited conformation of SmyD2 and restricts access to the catalytic site. Unexpectedly, despite that the two SmyD2 structures are highly superimposable, significant differences are observed in the first two helices of the CTDs: the two helices bend outwards and move away from the catalytic domain to generate a less closed conformation in the sinefungin-bound structure. Although the overall fold of the individual domains is structurally conserved among SmyD proteins, SmyD2 appear to be a conformational “intermediate” between a close form of SmyD3 and an open form of SmyD1. In addition, the structures reveal that the CTD is structurally similar to tetratricopeptide repeats (TPR), a motif through which many cochaperones bind to the heat shock protein Hsp90. Our results thus provide the first evidence for the intradomain flexibility of the TPR-like CTD, which may be important for the activation of SmyD proteins by Hsp90.

## Introduction

Covalent histone modifications represent an important regulatory mechanism controlling gene transcription, essential for normal growth and development [1]. Disrupting the balance of histone modifications can lead to the altered expression of genes involved in tumorigenesis including proto-oncogenes and cell cycle regulators [2]; however, little is known about how the enzymes that control histone modifications are regulated posttranslationally. Members of the SET and MYND domain containing (SmyD) family of proteins possess histone lysine methyltransferase capacity and have been shown to be involved in the transcriptional control of cell differentiation and cell proliferation [2], [3], [4], [5], [6]. The SmyD protein family consists of five proteins (SmyD1–5) that share about 30% sequence identity with each other and are grouped based on the presence of two conserved domains (MYND and SET domains) [4]. The MYND domain is a zinc finger motif that is involved in protein–protein interaction [7]. The SET domain is an evolutionarily conserved motif consisting of about 130 amino acids that is responsible for adding methyl groups to lysine residues of proteins using S-adenosylmethionine (AdoMet) as a donor substrate.

Evidence for a critical role of SmyD proteins during organ development was first shown by the constitutive knockout of SmyD1, resulting in early embryonic lethality due to disruption of cardiac differentiation and morphogenesis [3]. Subsequent reports have further indicated that SmyD proteins are indeed critical regulators of cardiac as well as skeletal muscle development [5], [8], [9], [10], [11]. Despite being highly expressed in heart and brain, a specific functional role for SmyD2 in these organs has not been well characterized [2], [10]. Overexpression of SmyD2 has been shown to cause changes in expression of genes associated with chromatin remodeling, cell cycle, and transcription regulation, indicating that this protein may function as a transcriptional regulator by methylating H3 K4 and participates in cell cycle regulation and cell growth [4]. Interest in SmyD2 has grown significantly because of recent reports indicating that SmyD2 repress transcriptional p53 activity by lysine methylation (Lys370), exerting an oncogenic and drug resistance action through inhibition of p53-mediated cell death pathways [12]. In addition to p53 methylation, a new study showed that the retinoblastoma tumor suppressor (RB), a central cell cycle regulator and tumor suppressor, can also be methylated by SmyD2 at lysine 860, which regulates the RB activity during cell cycle progression, cellular differentiation, and in response to DNA damage [13]. In agreement with these observations, SmyD2 recently has been shown to act as a cancer-promoting gene through activation or overexpression in esophageal squamous cell carcinoma [14]. These studies thus support a role for SmyD2 in the regulation of proliferation and in tumor progression, which underscores the importance of elucidating the regulation of SmyD2 activity.

The molecular chaperone Hsp90 plays an important role in the folding, activation, intracellular transport, and assembly of a broad range of client proteins, specifically chaperoning molecules involved in signal transduction and cell cycle regulation [15]. Mounting evidence showed that Hsp90 is also involved in transcriptional regulation and epigenetic inheritance by interacting with epigenetic proteins that function in chromatin remodeling and histone modifications [16], [17]. Based on the ability of Hsp90 to stimulate the activity of SmyD proteins, recent studies have characterized SmyD proteins as new clients of Hsp90 [4], [17]; however, the critical questions regarding how Hsp90 activates SmyD proteins remain poorly understood. Previous studies suggested that the methyltransferase activity of SmyD proteins is suppressed by an autoinhibited conformation maintained by the CTD, a helix bundle C-terminal to the catalytic SET domain that is conserved and unique in SmyD proteins [18], [19]. It has been proposed that the intra- and interdomain bending of the CTD may be central for the activation of SmyD proteins by Hsp90 [19]. In this paper, we report two crystal structures of full-length SmyD2 in complex with the methyltransferase inhibitor sinefungin (SFG) and the cofactor product S-adenosylhomocysteine (AdoHcy). Our studies demonstrate for the first time the intradomain flexibility of the CTD and reveal the structural resemblance of the autoinhibitory CTD to tetratricopeptide repeat (TPR) motif, which suggest a mechanism for the Hsp90-mediated activation of SmyD proteins. Our findings therefore contribute to the understanding of the mechanism that regulates the activity of SmyD proteins in early heart development and tumorigenesis.

## Results and Discussion

### SmyD2 structure with the TPR-like CTD

**Figure 1:**
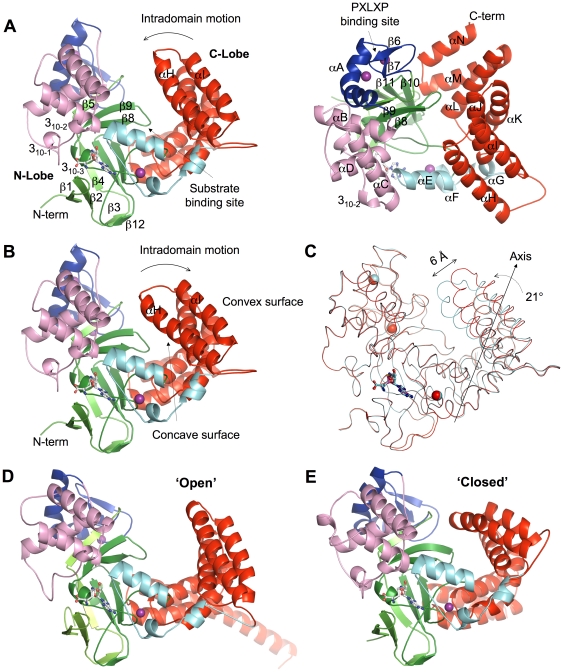
Ribbon diagram of the SmyD2 structures. (A) Side view (left) and top view (right) of the binary structure of SmyD2–sinefungin. (B) The structure of SmyD2–AdoHcy. Secondary structures of SmyD2, α-helices, 3_10_-helices, and β-strands are labeled and numbered according to their position in the sequence. The S-sequence, MYND, SET-I, core SET, post-SET, and CTD are depicted in light green, blue, pink, green, cyan, and red, respectively, while sinefungin and AdoHcy are represented by balls-and-sticks and zinc ions are denoted by purple spheres. (C) Superposition of two SmyD2 structures in complex with sinefungin (red) and AdoHcy (cyan) based on their N-lobes. The maximum distance between the equivalent regions in the outer edge of their C-lobes is indicated. The intradomain motion is indicated by the straight arrow and the approximate rotation angle is given. (D) Ribbon diagram of the structure of SmyD1 and (E) SmyD3 with the domains colored the same as above.

Two crystal structures of full-length SmyD2 in complex with the cofactor product AdoHcy and the methyltransferase inhibitor sinefungin have been determined at 2.1 Å and 1.8 Å by zinc single-wavelength anomalous dispersion (Table 1). Similar to SmyD1 and SmyD3 [18], [19], SmyD2 has a multidomain structure that folds into two lobes with overall dimensions of approximately 65 Å×40 Å×55 Å (Figure 1). Although the overall fold of their individual domains is structurally conserved, the SmyD family proteins differ dramatically in the relative orientation between the N- and C-terminal lobes. Detailed description of the structural differences will be addressed later in the article. The N-terminal lobe (residues 3–279) is composed of four domains: the catalytic SET domain, located in the middle of this lobe, is surrounded by the zinc finger MYND, insertion SET-I, and post-SET domains. Immediately C-terminal to the post-SET domain, the polypeptide forms a large domain of about 150 residues that constitutes the C-terminal lobe (residues 280–432). This domain is conserved in the SmyD proteins and was referred to as the CTD in our previous studies [18]. The CTD is composed of seven antiparallel α-helices (αH–αN) rotated relative to one another by an approximately 25°. This topology creates a right-handed superhelical structure generating a concave surface on one side with a convex surface on the other. Despite the absence of any significant sequence similarities, the overall fold of the CTD is reminiscent of that of TPR repeats that adopt a helix-turn-helix structure. Given that the TPR repeats mediate specific protein–protein interactions and the assembly of multiprotein complexes, the structural similarity of the CTD and the TPR repeats suggests a function for the CTD as a protein-protein interaction module.

**Table 1:** Crystallographic data and refinement statistics.

	Sinefungin	AdoHcy
Space group	P2_1_2_1_2_1_	P2_1_2_1_2_1_
Cell parameters (Å)		
a	57.5	57.9
b	75.1	75.0
c	112.5	113.4
Wavelength (Å)	0.97872	1.28215
Resolution (Å)	30.0-1.8	30.0-2.03
*R_merge_* a	0.083 (0.503)^b^	0.102 (0.512)
Redundancy	6.0 (5.8)	11.3 (10.3)
Unique reflections	45863	32539
Completeness (%)	99.7 (99.5)	99.9 (99.1)
〈I/σ〉	8.9 (2.8)	9.5 (4.6)
**Refinement**		
Resolution (Å)	30-1.8	30-2.03
Molecules/AU	1	1
*R_work_* c	0.186 (0.224)	0.173 (0.193)
*R_free_* d	0.208 (0.275)	0.215 (0.226)
RMSD		
Bond lengths (Å)	0.010	0.010
Bond angels (°)	1.0	1.1
No. of atoms		
Protein	3465	3453
Sinefungin/AdoHcy	27	26
Water	429	392
Zinc	3	3
B-factor (Å^2^)		
Protein	25.8	29.0
Sinefungin/AdoHcy	12.8	19.7
Water	33.4	35.5
Ramachandran plot		
Preferred regions (%)	97.12	96.92
Allowed regions (%)	2.88	3.08
Outliers (%)	0.0	0.0

a *R_merge_* = Σ|I−〈I〉|/ΣI, where I is the observed intensity and 〈I〉 is the averaged intensity of multiple observations of symmetry-related reflections.

b Numbers in parentheses refer to the highest resolution shell.

c *R_work_* = Σ|F_o_−F_c_|/Σ|F_o_|, where F_o_ is the observed structure factor, F_c_ is the calculated struture factor.

d *R_free_* was calculated using a subset (5%) of the reflection not used in the refinement.

**Figure 2:**
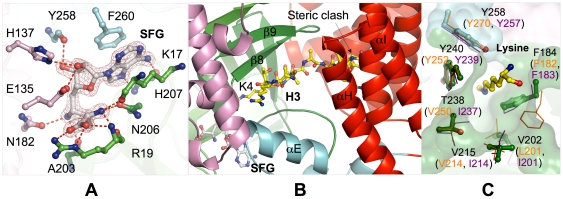
Cofactor binding pocket and substrate binding site. (A) Interaction between SmyD2 and sinefungin. SmyD2 residues are represented by balls-and-sticks with their carbon atoms colored according to the scheme in Figure 1. Sinefungin is depicted by balls-and-sticks overlaid with 2F_o_−F_c_ omit map calculated at 1.8 Å and contoured at 2.5 σ. Hydrogen bonds are illustrated as red broken lines. (B) Ribbon diagram of the putative substrate binding site, illustrating the interaction between SmyD2 and the modeled H3 peptide. The H3 peptide (1–10) from the Set7/9 structure (PDB code 1O9S) is displayed as balls-and-sticks with carbon atoms colored yellow. (C) Superposition of the target lysine-access channels of SmyD2, SmyD1, and SmyD3. The oval-shaped channel in SmyD2 is depicted by molecular surface. Residues in SmyD2 are represented by balls-and-sticks, while residues in SmyD1 and SmyD3 are displayed as sticks in purple and orange, respectively. Target lysine (H3K4) is colored in yellow.

The architecture of the catalytic SET domain of SmyD2 is essentially similar to that of SmyD1 and SmyD3 [18], [19], which features a “split” domain defined by two separated segments, the S-sequence (residues 3–49) and the core SET domain (residues 183–246). Despite the split in the primary structure, the SET domain in SmyD2 has the similar overall fold to other SET domain containing proteins, characterized by one central 3_10_ helix (3_10-3_) and 10 β-strands (β1–β5 and β8–β12) that are arranged into four antiparallel β-sheets (Figure 1). Of particular importance are the loop connecting 3_10-3_ and β10 that contributes conserved catalytic residues and functions to bind the cofactor at the bottom of the cofactor binding site, and the strand β8 and the loop following β10 that form a narrow cleft predicted to accommodate substrate H3 peptide (Figure 2). However, the SET domain alone is not sufficient for lysine methylation and it requires the cooperation with three other domains, including the N- and C-terminal flanking domains (pre-SET and post-SET) as well as the insertion SET-I domain [20], [21], [22]. The latter three domains are not conserved with highly variable structures in the known SET proteins but they occupy similar positions and play similar roles in these enzymes. Interestingly, SmyD2 does not contain the pre-SET domain, though this domain is required by other SET proteins to stabilize the SET domain fold or provide an extended histone binding site [21].

Both post-SET and SET-I domains are engaged in cofactor and substrate binding [20]. The post-SET domain, which is immediately downstream of the SET domain, is a small cysteine-rich region consisting of three short α-helices (αE, αF, and αG) that are organized around a single zinc ion (Figure 1). The zinc ion is coordinated by four highly conserved cysteine residues: Cys262, Cys264, and Cys267 from the post-SET domain and Cys209 from the SET domain. This zinc ion thus appears to be important for the folding of the post-SET domain and also tethers this domain to the SET domain. As a result of this tethering, the post-SET domain lies close to the active site, with the loop connecting αE and αF placed near the cofactor, and the C-terminal end of helix αE positioned to participate in the formation of the substrate binding cleft. Similar to other SmyD proteins [18], [19], SmyD2 has a large SET-I domain consisting of a helix bundle (αB, 3_10-1_, 3_10-2_, αC, and αD) of as many as 84 residues, together with the MYND inserted between the SET strands β5 and β8 (Figure 1). The equivalent region in Set7/9 or Dim-5, however, contains only one or two small helices of 15–20 residues [23], [24]. In contrast to the MYND, the SET-I domain packs against the opposite face of the β-sheet containing β4, β10, and β11, contributing to the cofactor and substrate binding. Specifically, the last two helices (αC and αD) of the SET-I domain might be important for the recognition of the H3 N-terminal residues (Figure 2), while the loop between the 3_10-1_ and 3_10-2_ helices makes extensive contacts with the cofactor.

MYND mediates protein–protein interactions by binding to a proline-rich sequence [7]. It has been demonstrated that the MYND present in SmyD2 interacts with proteins containing the PXLXP motif, such as EBP41L3, a functional suppressor of epithelial ovarian cancers [4]. As shown in Figure 1A, the MYND consists of one kinked α helix (αA) and two antiparallel β-strands (β6, β7) that are organized around 2 zinc ions. Despite that it forms direct contacts with the catalytic SET domain, the MYND does not contribute residues to cofactor binding. In addition, this domain is more than 10 Å away from the putative substrate-binding pocket and may not be directly involved in substrate recognition (Figure 2). These observations are in agreement with previous findings that the MYND is dispensable for the histone methylation activity of SmyD2 [4], implicating that the MYND may primarily function as a protein–protein interaction module and cooperate SmyD2 with other proteins to regulate tumor proliferation and progression. The structure of the MYND of SmyD2 is very similar to that of SmyD1, SmyD3 and AML1/ETO [7], [18], [19], with the following pairwise RMSDs for Cα atoms over 40 residues: 0.48 Å, 0.53 Å, and 0.81 Å, respectively. Superposition of the MYNDs of SmyD2 and AML1/ETO, which was solved in complex with a peptide containing the “PPPLI” motif [7], reveals that the proline-rich peptide is located in a shallow, fully exposed surface groove that is readily accessible by other proteins. One side of the groove is formed by a loop connecting β6 and β7, and the other side by the residues from the N-terminal half of helix αA. Three highly conserved residues (Trp80, Gln76, and Tyr70 in SmyD2) critical for AML1/ETO binding to the peptide are highly superimposable in the two structures. The high structural similarity suggests a similar mode of recognition of proline-rich sequences shared by these two MYNDs.

### Active site characterized by a spacious target lysine access channel

We have determined two complex structures of SmyD2 bound either to the cofactor product AdoHcy or to a potent methyltransferase inhibitor sinefungin. The two structures are remarkably similar to each other in terms of cofactor binding. Therefore, the following discussion on the interaction between SmyD2 and the cofactor will be solely focused on the SFG-bound SmyD2 structure. Similar to that in other SmyD proteins [18], [19], the L-shaped sinefungin binds in a deep surface pocket formed by the SET-I, SET and post-SET domains (Fig. 2A). In particular, the adenine moiety of sinefungin is sandwiched between the benzyl ring of Phe260 and the aliphatic side chain of Lys17, with its purine N6 and N7 atoms hydrogen-bonding to the backbone carbonyl and amide groups of His207, respectively. The ribose hydroxyls of the cofactor make three hydrogen bonds with the side chains of His137 and Glu135 and the carbonyl oxygen of Tyr258. At the opposite end of sinefungin, the positively charged α-amino group is recognized by a trigonal array of hydrogen bonds with the main chain carbonyl oxygens of Lys17 and Arg19 and the amide Oδ of Asn206, while the carboxylate moiety forms salt-bridge interactions with the guanidinium group of Arg19. The latter electrostatic interactions are present in most SET proteins including SmyD1 but are replaced by a hydrogen bond to a tyrosine residue in SmyD3, which represents an unusual variation [19]. In the middle of sinefungin, the C–NH3 amine group, which is in place of the S–CH3 sulfonium of AdoMet, engages in two hydrogen bonds with the backbone oxygen of Ala203 and the amide Oδ of Asn182. The similar interactions are expected in the case of AdoMet, which might contribute to enzymatic function by destabilizing the active methyl group. Collectively, the overall cofactor-binding mode of SmyD2 is structurally conserved with SmyD1 and SmyD3 and other SET enzymes and serves to orient the methyl group of AdoMet into the methyltransfer pore during catalysis.

Although the SmyD2 structures were solved without substrate, superposition of SmyD2 with histone H3-bound Set7/9, a H3 lysine 4 methyltransferase, offers insights into substrate recognition. As shown in Figure 2B, the modeled H3 peptide binds in a deep, rectangle-shaped cleft formed by the SET, post-SET and SET-I domains. In the cleft, the β8 strand and the loop preceding the post-SET domain are predicted to interact with substrate histone in a hybrid β-sheet-binding mode as shown in other SET proteins [20]. Lys4 of the peptide is at the center of this β-sheet interaction, which firmly deposits its side chain into the target lysine access channel that leads to sinefungin that binds on the opposite face of the SET domain. Comparison of SmyD2, SmyD1, and SmyD3 reveals that the structures of the lysine access channel of these enzymes are similar to each other with a large oval-shaped opening (Figure 2C). The residues in SmyD2 involved in the formation of the channel including Tyr240, Tyr258, Val202, Val215, and Thr238, are highly structurally aligned with the equivalent residues in SmyD1 and SmyD3, except for Phe184. The spacious lysine access channel is a characteristic feature of SmyD proteins, which is mainly attributed to the replacement of some bulky aromatic residues in Set7/9 or other SET proteins by small hydrophobic ones in SmyD proteins [18], [19]. In SmyD1, substitution of Val214 by tyrosine, a mutation that would create a tighter active site pocket, results in a significant increase in H3 binding and also enhances SmyD1 methylation, indicating that this large channel made SmyD1 unable to effectively interact with the target lysine during methyl transfer, affecting its enzymatic activity [18].

A unique feature of SmyD proteins is the presence of the conserved CTD, which is located near the substrate binding cleft and together with the SET domain forms a deep canyon that spans the entire molecule [18], [19]. Similar to SmyD3, the putative substrate binding site of SmyD2 is located at the bottom of the 15-Å-deep crevice, with the CTD acting like a lid and partially covering the active site pocket (Figure 1). However, because of the location of the CTD, severe steric clashes are observed between the C-terminus of the peptide and the CTD inner surface in the SmyD2–H3 model (Figure 2B). The steric hindrance of the CTD suggests that the CTD prevents H3 binding and it may be required to move away to allow substrate entry and efficient catalysis. Alternatively, this might be an indication that the H3 peptide may adopt a different conformation when binding to SmyD2. Considering the potential motion of the CTD, it is also likely that the CTD conformation observed in the crystal structures represents a non-physiological state of the protein. Importantly, mutation or deletion of the CTD significantly increased both substrate binding and H3 methylation by SmyD1, demonstrating that this domain plays a negative role in the regulation of the protein's activity [18]. Together with previous functional studies [4], [18], these observations support the idea that the histone methyltransferase activity of SmyD proteins is regulated by autoinhibition that involves the conserved CTD.

### Maintenance of SmyD2 autoinhibited conformation

**Figure 3:**
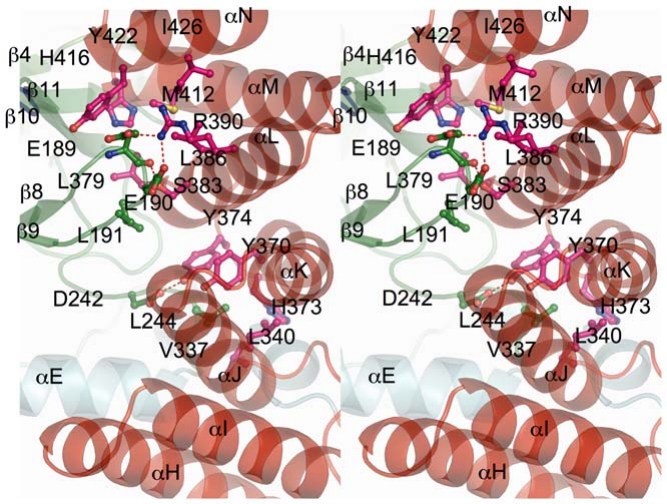
Stereo view ribbon diagram of the domain interface of N- and C-terminal lobes. Residues are colored according to domain in which they reside, and hydrogen bonds are indicated as red dashed lines.

SmyD2 methylates histone H3 to a very limited extent both *in vitro* and *in vivo* [4], and extensive interactions between the CTD and the SET domain appear to contribute to the maintenance of the autoinhibited state of SmyD2 (Figure 3). Specifically, the interactions involve contacts mediated by the turns connecting the CTD helices, which form a contiguous ridge that is anchored to the concave face of the β-sheet containing β4, β10, and β11. In addition, the residues within the antiparallel β-hairpin between β8 and β9 appear to play a central role in the interaction with the CTD. This hairpin protrudes deep into the middle of the concave face of the CTD, braced by the CTD helices and forming numerous direct interactions with αH, αL, αM, and αN. In contrast, the equivalent hairpin in SmyD1 interacts only with the last helix (αN) from the CTD, separated by a large crevice from the other CTD helices. In particular, the aliphatic side chain of Glu189 stacks with the aromatic ring of Tyr422, together with residues Leu191, Leu379, Leu386, Met412, and Ile426 forming a continuous hydrophobic core that stretches from the hairpin down to the bottom of the domain interface. Of particular importance, however, are hydrogen bonds formed between with the guanidinium group of Arg390, which projects from helix αL, and two acidic residues, Glu189 and Glu190 in the β8–β9 hairpin. A similar interaction between the β8–β9 hairpin and the CTD was also observed in SmyD3 but absent in SmyD1 that has an open conformation [18], [19]. Given that the β8–β9 hairpin makes extensive contacts with the CTD, this hairpin is likely to be important in holding the SET domain and the CTD together and maintaining the closed conformation of the substrate binding cleft.

Additional interactions that participate in stabilizing the closed conformation are made among the residues in the loop preceding the post-SET domain and residues in the third and fourth helices (αJ and αK) of the CTD (Figure 3). Specifically, Asp242 forms a hydrogen-bond interaction with Tyr374, while Leu243 participates in a hydrophobic cluster with Val337, Leu340, Tyr370, His373, and Tyr374 from the CTD. Most of these residues are well conserved in SmyD family proteins [18], suggesting that the interactions between them may also contribute to the maintenance of the autoinhibited state. Interestingly, substitution of the corresponding Asp242 or Tyr370 by alanine is able to destabilize the autoinhibited state of SmyD1, leading to a significant increase in both H3 binding and the enzymatic activity [18].

### Intradomain and interdomain flexibility of the conserved CTD

**Figure 4:**
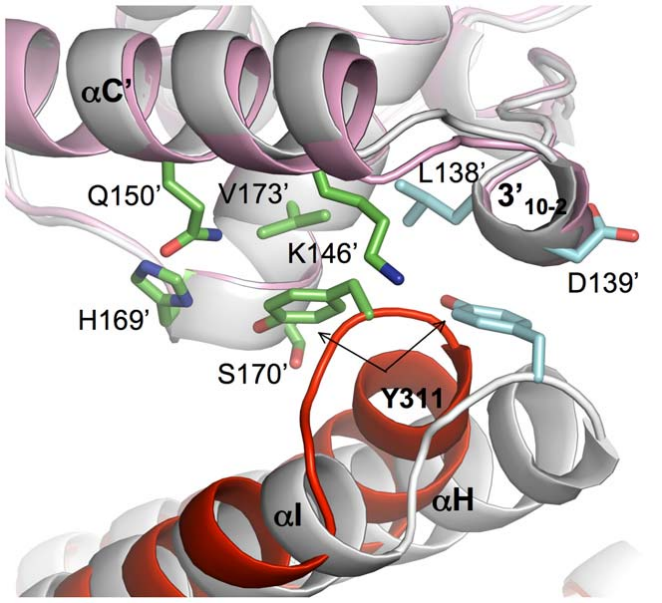
Comparison of the crystal contacts of SmyD2–SFG and SmyD2–SAH. SmyD2–SFG is depicted in red and its symmetry-related molecule in pink. Both SmyD2–SAH molecules are colored gray. Residues involved in crystal contacts are displayed as sticks and colored green and cyan in SmyD2–SFG and SmyD2–SAH, respectively. The prime symbol denotes residues and secondary structures in the symmetry-related molecules.

The intra- and interdomain bending of the CTD has been proposed to be central to the release of the autoinhibitory effect exerted by the CTD [19]. However, there has been no direct evidence to support this model. Significantly, the two SmyD2 structures in complex with the cofactor analogs sinefungin and AdoHcy differ dramatically in the conformation of the CTD (Figure 1C). Despite that the structures of SmyD2–SFG and SmyD2–AdoHcy are highly superimposable with RMSD of 0.36 Å over 400 residues, close examination reveals that the first two helices of the CTD (αH and αI) adopt different conformations. These two helices bend outwards with the loop between the two helices moving ∼6 Å further away from the catalytic SET domain. This motion generates a less closed conformation in the SFG-bound SmyD2 structure and slightly tightens the cavity of the active site in SmyD2–AdoHcy. In agreement with the conformational changes, the flexible nature of the αH and αI helices is also indicated by their higher than average isotropic temperature factors of 41.9 Å^2^ for SmyD2–AdoHcy and 39.5 Å^2^ for SmyD2–SFG (Table 1). We use the program DynDom to further analyze this domain movement [25]. Two hinge bending motion regions are identified as containing residues 294–300 and 319–322, at which point the αH and αI helices pivot towards the SET domain by the rotation. The hinge axis of the rotation runs approximately perpendicular to the axis of the CTD superhelix, intersects the helix αH and is located 1.4 Å from Cα of Arg299 and 1.3 Å from Cα of Asn300. The translation component of the screw operation describing the domain movement is 1.5 Å so that the movement is essentially a pure domain rotation. The crystal packing constraints appear to help to stabilize the conformational diversity of SmyD2. The overall crystal packing is effectively identical in the SmyD2–AdoHcy and SmyD2–sinefungin complexes, except at the packing interfaces that involve αH and αI (Figure 4). The different orientations of these two helices are stabilized by the differences in crystal packing contacts that contain spatially close but distinct sets of residues. Collectively, these findings provide the first evidence of the intradomain flexibility of the CTD and the structural basis for the model of the conformational changes in the CTD that regulates the activity.

The CTD is located over 30 Å distant from the cofactor and does not contribute residues to cofactor binding. It is not apparent from the structure how such a long-range conformational change is triggered by the cofactors and propagated from the cofactor binding pocket, because of the highly superimposable cofactor binding sites and no significant structural changes in their immediate neighboring regions. There are, however, some differences caused by the CTD motion in the interaction networks between the CTD and post-SET domain, including a new hydrogen bond between the side chains of Arg299 and Glu248 and the potential salt-bridge interactions between Arg306 and Asp256 in SmyD2–AdoHcy. Nevertheless, the long-range conformational change triggered by the exchange of the cofactors could have at least one important functional implication. Sinefungin more resembles AdoMet than AdoHcy in structure, with the C–NH3 amine group in place of the S–CH3 sulfonium. Our findings may then suggest that the binding of the substrate AdoMet to SmyD2 may partially relieve the inhibition by the CTD by causing it to move away from the catalytic domain. The ability of the conformation changes induced by cofactors appears to be specific to SmyD2. Several structures of SmyD3 have been recently been deposited in the protein data bank including SmyD3–AdoMet, SmyD3–sinefungin, and SmyD3–AdoHcy complexes [19], [26]. Despite marked differences in crystal packing, these SmyD3 complexes display essentially identical structures independent of the types of cofactor, suggesting some differences in allosteric properties among SmyD family members.

The exceptionally large differences in the domain-domain orientation or with respect to the distance separating the N- and C-terminal lobes have been observed between SmyD1 and SmyD3 [18], [19]. As a result of the differences, the CTD in SmyD3 adopts a closed conformation that blocks the putative H3K4 binding cleft, whereas the SmyD1 CTD displays an open state with the active site completely exposed. Interestingly, the SmyD2 structures display substantial differences from both SmyD1 and SmyD3 in regard to the CTD orientation. The differences can be viewed when the N-terminal lobes from SmyD2, SmyD1, and SmyD3 are structurally aligned as shown in Figure 1. In this view, the N-terminal lobe remains essentially unchanged, but the CTDs move to either widen or narrow the deep crevice between the N- and C-terminal lobes, essentially mimicking how a clam shell opens and closes. In particular, the Cα atoms of some residues near the outer edge of the CTD move as much as 12 Å between SmyD3 and SmyD1, whereas two SmyD2 structures appear to be a conformational “intermediate” between the close form of SmyD3 and the open form of SmyD1. Although the active site pocket of both SmyD2 and SmyD3 is partially closed by the CTD that leads to steric clash with the modeled H3 peptide, significant differences are observed in the first two helices of the CTD. The helices equivalent to αH and αI in SmyD3 form direct contact with the linker region between the SET and MYND domains [19], but the SmyD2 structures reveal that these two helices swing outwards and maintain a narrow gap with the SET domain on top of the active site pocket. This structural difference, however, does not cause a significant change in the contact area between the CTD and the rest of protein, with the total buried surface area in the domain interface of 3766 Å^2^ in SmyD3 compared to 3796 Å^2^ and 3682 Å^2^ in SmyD2–AdoHcy and SmyD2–SFG, respectively. Taken together, the differences in the domain–domain orientation between SmyD2 and other SmyD proteins further suggest that the CTD is able to undergo a hinge bending-like motion, which could regulate access to the active site.

### A model of SmyD2 activation by Hsp90

It has been reported that interaction between SmyD2 and Hsp90 is important for the histone methyltransferase activity of SmyD2, which is in agreement with results for SmyD1 and SmyD3 [4], [5], [6]. The manner in which Hsp90 contributes as a cofactor of SmyD proteins is still unclear. Given the differences in the CTD conformations of SmyD proteins, it has been proposed that Hsp90 activates SmyD proteins through the displacement of the autoinhibitory effect of the CTD, which in turn leads to the exposure of the CTD-blocked active site [19]. However, the question regarding the mechanics of how Hsp90 causes the CTD motion remains elusive. Hsp90 is essential for maintaining the activity of numerous signaling proteins and it plays a key role in cellular signal transduction networks [27]. In fulfilling its role, Hsp90 often operates by interacting with a variety of proteins that contain a TPR domain. At the very C-terminal end of Hsp90 is the TPR motif recognition site, a conserved MEEVD pentapeptide, that is responsible for the interaction with many TPR proteins such as the immunophilins FKBP51/52, the stress induced phosphoprotein Hop, cyclophilin Cyp40, and a protein phosphatase PP5 [28].

**Figure 5:**
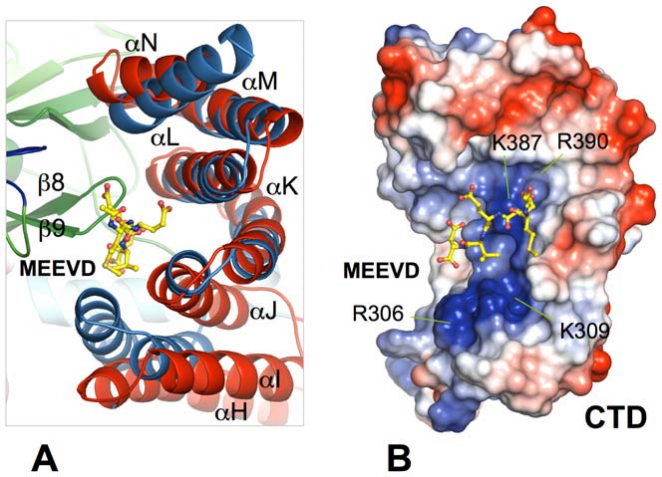
TPR-like CTD. (A) Superposition of the CTD of SmyD2–SFG (red) and the TPR2 domain of Hop (sky blue) (PDB code 1ELR). The Hsp90 MEEVD peptide in complex with the Hop TPR2 domain is displayed as balls-and-sticks with carbon atoms colored yellow. (B) Model of the Hsp90 MEEVD peptide bound in the SmyD2 CTD. The CTD is represented by molecular surface with color coding according to the electrostatic potential: red, white, and blue correspond to negative, neutral, and positive potential, respectively, whereas the peptide is shown as balls-and-sticks. Positively charged residues predicted to be essential for peptide binding are labeled.

Interestingly, a search using the Dali server reveals that the conserved CTD, which sterically blocks the substrate binding site, resembles the structure of TPR repeats [29]. The CTD is mainly comprised of three copies of 34-amino acid, helix-turn-helix TPR motifs, including αH–αI, αJ–αK, and αL–αM. As shown by superposition of the CTD of SmyD2 and the TPR2 domain of Hop, the overall configuration of these two domains are similar to each other with RMSD of 3.9 Å over 128 Cα atoms (Figure 5A). The only significant difference is the first two helices of the CTD (αH and αI), which have a different degree of superhelical twists. The structural similarity of the CTD and TPR repeats leads us to hypothesize that the CTD might interact with Hsp90 via the C-terminal MEEVD pentapeptide of the chaperone, which may be important for SmyD2 activation. This hypothesis is in agreement with previous studies showing that Hsp90 interaction with SmyD2 was mediated through a region other than the MYND and SET domains [4]. To assess potential interaction between the CTD and Hsp90, we performed a modeling study using the structure of the TPR2 domain of Hop in complex with a C-terminal pentapeptide MEEVD of Hsp90 (Figure 5A). In the structure of the Hop–MEEVD complex, the Hsp90 peptide interacts with the Hop TPR2 domain in an extended conformation, with the peptide sequence running parallel with the helices of the TPR motifs [30]. The peptide-protein interactions are primarily dominated by hydrogen bonds and salt-bridges involving the carboxylated groups of acidic residues and the C-terminus of the Hsp90 MEEVD motif interacting with conserved arginine and lysine residues lining the basic peptide binding channel of Hop. Despite the low sequence identities of 16% between the CTD and the TPR2 domain, most of the arginine, lysine and asparagine residues responsible for Hop–Hsp90 interactions are structurally conserved in SmyD2, including residues Arg306, Lys309, Gln345, Lys387, and Arg390. By lining up along the concave surface of the CTD, these residues create a continuous positively charged groove predicted for engagement of the Hsp90 acidic C-terminal region (Figure 5B). This putative MEEVD binding site, however, is partially buried and occupied by the loop between strands β8 and β9, the region that is involved in maintaining the autoinhibited state of the protein by interacting with the CTD (Figure 5A). The structural similarity of the CTD to the TPR2 domain together with the buried MEEVD binding site may suggest a mechanism of SmyD2 activation by Hsp90, which may resemble how PP5 is activated by Hsp90. The crystal structure of autoinhibited PP5 reveals that the TPR domain of PP5 engages with the catalytic channel of the phosphatase domain, restricting access to the catalytic site [31]. This autoinhibited conformation of PP5 is stabilized by the C-terminal helix that contacts a region of the Hsp90-binding groove on the TPR domain. Hsp90 activates PP5 by disrupting TPR–phosphatase domain interactions, permitting substrate access to the constitutively active phosphatase domain. Based on these analyses, we propose a model of SmyD2 activation by Hsp90, in which the Hsp90 MEEVD motif could compete with the β8–β9 hairpin for binding to the SmyD2 CTD, displacing the CTD from the substrate binding site and causing a conformational change in the CTD. This model is in agreement with the conformational flexibility of the CTD as revealed by the structural differences between SmyD2–AdoHcy and SmyD2–SFG (Figure 1). Additional research is required to support this proposed mechanism and to determine whether Hsp90 interacts with the SmyD2 via the CTD and induces a conformational change in this domain.

## Materials and Methods

### Protein Preparation

Protein purification was performed essentially as described previously [18]. Briefly, mouse SmyD2 was cloned into the pSUMO vector (LifeSensors), with an N-terminal His6-SUMO tag. Recombinant SmyD2 was then transformed into *Escherichia coli* for protein expression. The transformants were grown to an OD_600_ (optical density at 600 nm) of 0.4 at 37°C in 2 L LB medium, and then induced with 0.1 mM isopropylthio-β-D-galactoside at 15°C overnight. The cells were harvested, and lysed by French Press. The soluble fraction was then subjected to a series of chromatography purification by an AKTA purifier system (GE healthcare), and His6-SUMO tag was cleaved off with yeast SUMO Protease 1. SmyD2 proteins were finally purified to apparent homogeneity and concentrated to 10–20 mg/ml in 20 mM Tris–HCl (pH 8.0), 150 mM NaCl, 1 mM β-mercaptoethanol, and 5% glycerol.

### Crystallization and data collection

Prior to crystallization, SmyD2 (10 mg/ml) was incubated with 2 mM AdoHcy or sinefungin at 4°C for 2 h. The binary complex of SmyD2–AdoHcy or SmyD2–sinefungin was then crystallized by hanging drop vapor diffusion at 20°C, with 15% polyethylene glycol 8000, 50 mM NaCl, 100 mM Tris, pH 8.0. Crystals typically appeared within 1 day, achieved their full size in a week. X-ray diffraction data from single crystals were collected at beamline 21IDD at the Advanced Photon Source (Argonne, IL) and were then processed and scaled using the program HKL2000 [32]. The crystals belong to the orthorhombic space group P2_1_2_1_2_1_ and contain one molecule in the asymmetric unit (Table 1).

### Structure determination and refinement

The crystal structure of SmyD2 in complex with AdoHcy was solved by the single-wavelength anomalous diffraction (SAD) method using three intrinsic zinc ions. Initial phases were obtained using the program SOLVE [33], which was able to identify all three zinc sites with a figure of merit of 0.329 in the resolution range 20–2.1 Å. After density modification with the program RESOLVE [33], the resulting electron density map is interpretable. With the modified phases, automated model building was carried out by RESOLVE, which built 80% of the protein residues including side chains. The model was then completed and improved by alternating cycles of manual model building and refinement using COOT [34] and BUSTER [35]. The final refined model is well ordered with the exception of the first two residues and the last residue. Because of isomorphism of crystals (Table 1), the crystal structure of SmyD2 in complex with sinefungin was solved by rigid-body fitting of the SmyD2−AdoHcy model followed by manual model building and refinement as described above. The final models were analyzed and validated with PROCHECK [36]. All figures of 3D representations of the SmyD2 structures were made with PyMOL (www.pymol.org).

### Protein Data Bank accession number

Coordinates and structure factors have been deposited in the Protein Data Bank with accession number 3QWV and 3QWW for SmyD2−AdoHcy and SmyD2−SFG, respectively.
